# Inhibition of Methyltransferases Accelerates Degradation of cFLIP and Sensitizes B-Cell Lymphoma Cells to TRAIL-Induced Apoptosis

**DOI:** 10.1371/journal.pone.0117994

**Published:** 2015-03-04

**Authors:** Frank K. Braun, Rohit Mathur, Lalit Sehgal, Rachel Wilkie-Grantham, Joya Chandra, Zuzana Berkova, Felipe Samaniego

**Affiliations:** 1 Department of Lymphoma and Myeloma, The University of Texas MD Anderson Cancer Center, Houston, Texas, United States of America; 2 Department of Pediatrics, The University of Texas MD Anderson Cancer Center, Houston, Texas, United States of America; 3 Sanford-Burnham Medical Research Institute, La Jolla, California, United States of America; IISER-TVM, INDIA

## Abstract

Non-Hodgkin lymphomas (NHLs) are characterized by specific abnormalities that alter cell cycle regulation, DNA damage response, and apoptotic signaling. It is believed that cancer cells are particularly sensitive to cell death induced by tumor necrosis factor α–related apoptosis-inducing ligand (TRAIL). However, many cancer cells show blocked TRAIL signaling due to up-regulated expression of anti-apoptotic factors, such as cFLIP. This hurdle to TRAIL’s tumor cytotoxicity might be overcome by combining TRAIL-based therapy with drugs that reverse blockages of its apoptotic signaling. In this study, we investigated the impact of a pan-methyltransferase inhibitor (3-deazaneplanocin A, or DZNep) on TRAIL-induced apoptosis in aggressive B-cell NHLs: mantle cell, Burkitt, and diffuse large B-cell lymphomas. We characterized TRAIL apoptosis regulation and caspase activation in several NHL-derived cell lines pre-treated with DZNep. We found that DZNep increased cancer cell sensitivity to TRAIL signaling by promoting caspase-8 processing through accelerated cFLIP degradation. No change in cFLIP mRNA level indicated independence of promoter methylation alterations in methyltransferase activity induced by DZNep profoundly affected cFLIP mRNA stability and protein stability. This appears to be in part through increased levels of cFLIP-targeting microRNAs (miR-512-3p and miR-346). However, additional microRNAs and cFLIP-regulating mechanisms appear to be involved in DZNep-mediated enhanced response to extrinsic apoptotic stimuli. The capacity of DZNep to target cFLIP expression on multiple levels underscores DZNep’s potential in TRAIL-based therapies for B-cell NHLs.

## Introduction

Non-Hodgkin lymphomas (NHLs), a highly heterogeneous group of lymphoproliferative neoplasms, were the eighth most prevalent cancer in the United States and the sixth most prevalent cancer in U.S. males in 2010. Three types of aggressive B-cell NHLs responsible for early death of afflicted individuals are diffuse large B-cell lymphoma, mantle cell lymphoma, and Burkitt lymphoma, which account for 30%-40%, 5%, and 1%-2% of NHLs, respectively [17, 20, 29, 43]. The survival of individuals with NHL has improved with the addition of targeted therapies to conventional chemotherapy regimens. However, despite the use of targeted therapy and chemotherapy, NHLs show frequent relapses [38, 53]. Even the recently approved drugs for relapsed NHL, temsirolimus, bortezomib and ibrutinib, show only incremental improvement and patients still face an expected 5 year survival slightly above 50%. Thus, additional new targets and approaches to improve the efficacy of NHL therapy are urgently needed [57].

Defects in apoptotic signaling are one of the cancer hallmarks[19] and correlate with the aggressive behavior of relapsed NHLs and their resistance to chemotherapy. Activation of the extrinsic apoptotic pathway is the key element of responses to many commonly used cancer therapies [35]. Extrinsic apoptotic pathway signaling is initiated by the binding of death ligands (including tumor necrosis factor α–related apoptosis-inducing ligand [TRAIL] and FasL/CD95) to their respective death receptors (DR4, DR5, and Fas, respectively), prompting the formation of the death-inducing signaling complex and subsequent activation of caspase-8, which triggers a caspase cascade, culminating in DNA fragmentation and cell death [24]. Important inhibitors of apoptotic signaling are the long and short isoforms of cFLIP (cFLIP_L_ and cFLIP_S_) [40].

TRAIL is well known for its tumor-specific cytotoxicity. Several pre-clinical trials have investigated the potential of TRAIL-based therapies for NHLs. However, those therapies showed only modest activity as single-agents, and no TRAIL receptor-targeting therapy has been approved by the U.S. Food and Drug Administration to date [4, 18]. TRAIL signaling is often impaired in cancer cells, and this hurdle to TRAIL tumor cytotoxicity might be overcome by combing TRAIL-based therapy with drugs that reverse blockages of its apoptotic signaling.

Hypermethylation is associated with gene silencing and part of regulation of signaling pathways [32] and correlates with aggressive tumor growth and poor clinical outcome [7, 45]. Epigenetic modifications evidently play a crucial role in maintenance, development and pathogenesis of hematologic malignancies[47] and overexpression (e.g. EZH2), fusion proteins (e.g. MLL-DOT1L) and genetic alterations of methyltransferases are observed in several lymphomas [9, 39, 42, 46]. This indicates that inhibition of methyltransferase activity is a viable approach to target lymphoma biology [54] and therapies aiming at modulating epigenetic features have shown efficacy in hematopoietic cancers [28, 50]. However, azacitidine and decitabine, which irreversibly inhibit the DNA methyltransferase enzymes DNMT1 and DNMT3, are currently the only available FDA approved epigenetic drugs [22, 55]. We hypothesized that TRAIL-based therapy aiming to restore apoptosis in NHLs could benefit from the combination with pan-methyltransferase inhibitors [26].

3-deazaneplanocin A, a pan-methyltransferase inhibitor also known as DZNep, has been shown to remove histone 3 hypermethylation marks associated with gene silencing and to increase cell death in combination with histone deacetylase inhibitors [11, 14, 27, 31]. In this study, we investigated the impact of DZNep on TRAIL-induced apoptosis and found that DZNep accelerates cFLIP degradation, and thus enhances TRAIL-induced apoptosis in cell lines derived from various types of B-cell lymphoma.

## Results

### DZNep inhibits growth of lymphoma cells and enhances their sensitivity to TRAIL-induced apoptosis

**Fig 1:**
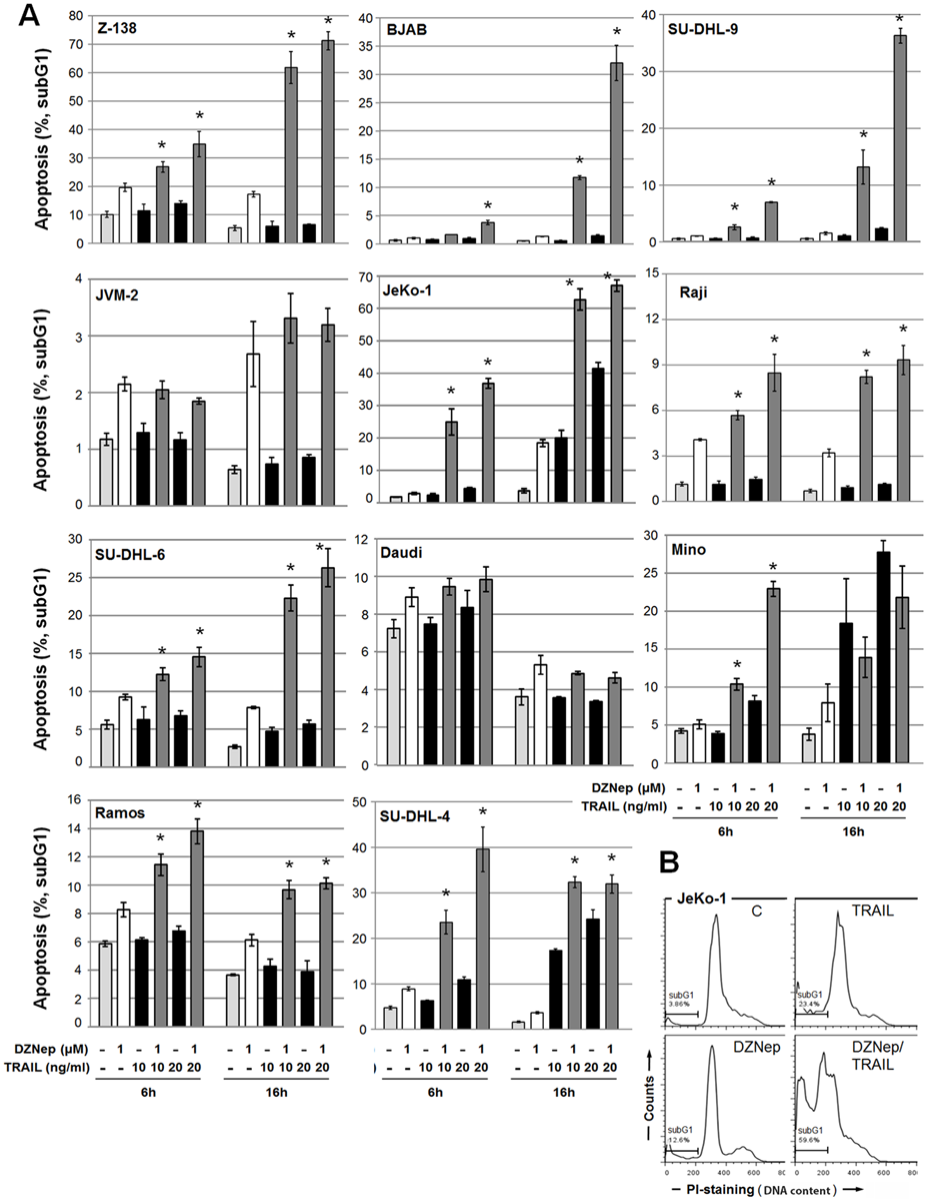
Inhibition of methyltransferases by DZNep sensitizes B-cell lymphoma cells to TRAIL-mediated apoptosis. (**A**) Cells were pre-treated with DZNep (1 μM) for 24 h followed by stimulation with TRAIL (10 or 20 ng/ml) for 6 or 16 h. Apoptosis was determined according to DNA fragmentation by propidium iodide staining (subG1 analysis). (**B**) The subG1 population for JeKo-1 cells is indicated in representative histograms of propidium iodide-stained cells.

**Fig 2:**
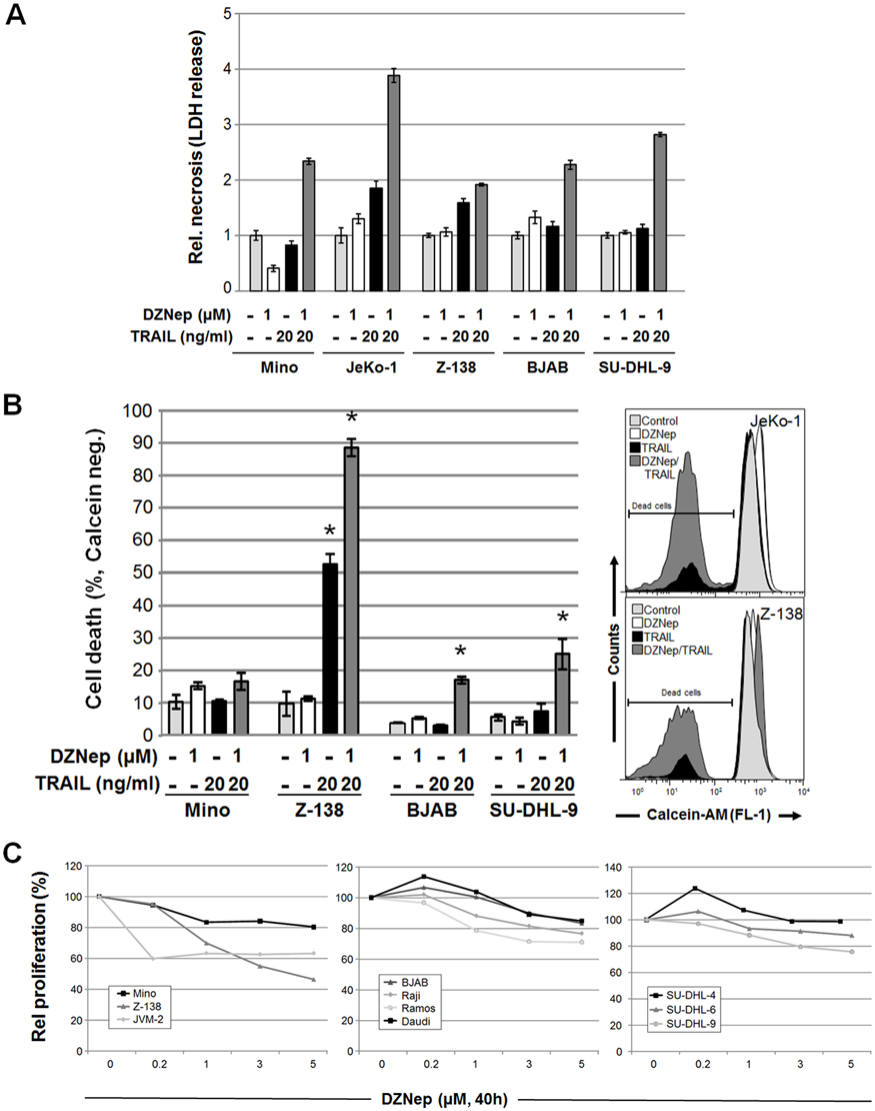
DZNep impairs cell growth of B-cell lymphoma cells. (**A**) Necrosis of cells treated as indicated was determined according to lactate dehydrogenase release in the supernatant. (**B**) Cell viability was determined by calcein AM staining (esterase activity analysis) of cells treated as indicated, and the apoptotic cell population (reduced esterase activity) is indicated in representative histograms. (**C**) Cells were incubated with DZNep (0.2, 1, 3, or 5 μM) alone for 40 h, and cell proliferation was determined by CellTiter 96 assay.

To test whether DZNep affects TRAIL signaling in various NHL B-cell lymphoma-derived cell lines, we investigated apoptosis induced by treatment with TRAIL in cells pre-treated with DZNep. This pre-treatment significantly enhanced TRAIL-induced apoptosis as determined by DNA fragmentation (subG1 cell population) in all but JVM-2 and Daudi cell lines (Fig. 1A-B). In addition apoptosis induction by DZNep alone was also not specific for any NHL group. Using agonistic ligands specific to TRAIL receptors DR4 and DR5, we found that DZNep-pre-treatment enhanced cell killing through both receptors (data not shown). Analysis of supernatant LDH levels showed no significant role of necrosis in observed cell death (Fig. 2A). However, DZNep- and TRAIL-treated cells showed less viability than that shown by cells treated with TRAIL alone (Fig. 2B).

Treatment of eleven cell lines, representing different NHL types, with increasing doses of DZNep for 40 h variably but significantly decreased cell proliferation in all cell lines compared with results after buffer treatment (Fig. 2C). This result shows clear cell death and anti-proliferative effects of DZNep pre-treatment.

### TRAIL-induced caspase activation is enhanced and accelerated in DZNep-pre-treated cells

**Fig 3:**
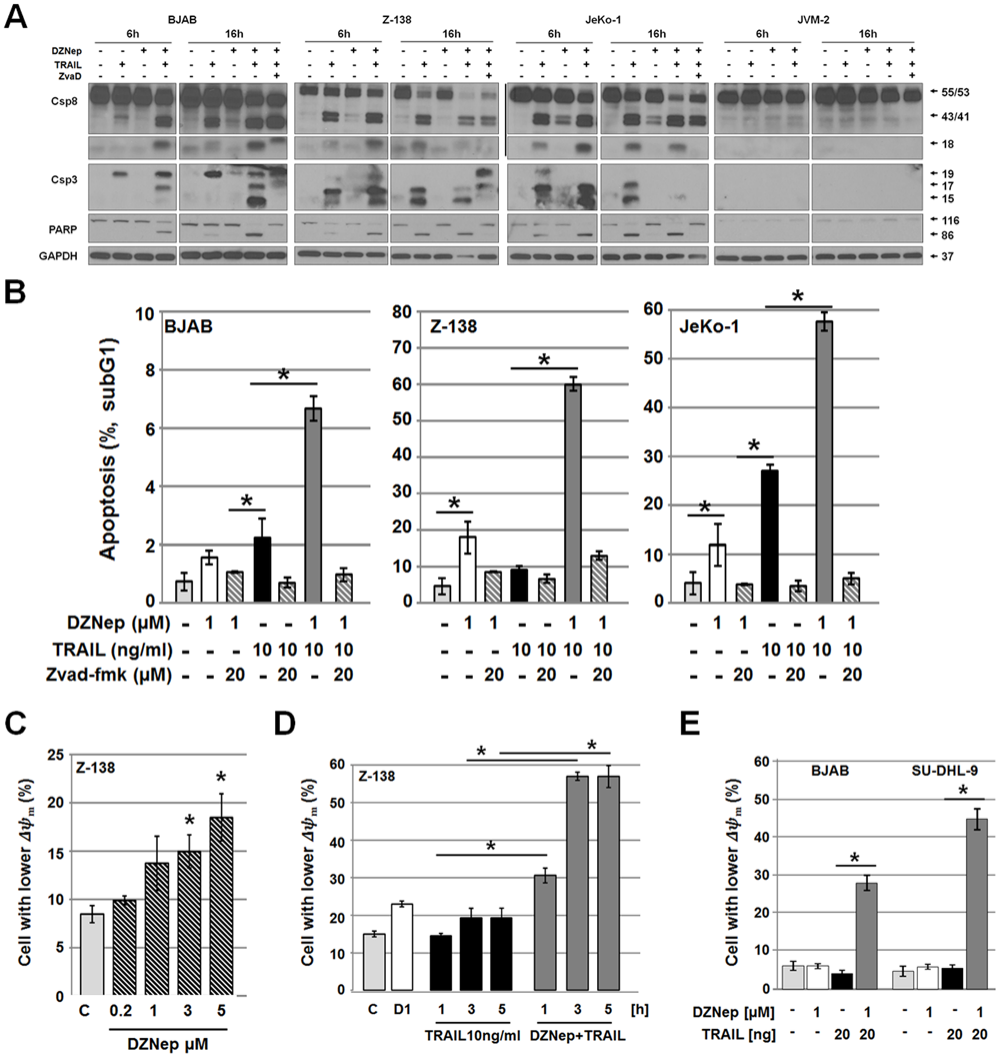
DZNep-mediated sensitization to TRAIL correlates with enhanced activation of caspase signaling and involvement of the mitochondrial apoptosis pathway. (**A**) Analysis of caspase-8, caspase-3, and PARP processing of cells pre-treated with DZNep (1 μM, 24 h) followed by TRAIL (10 or 20 ng/ml) stimulation for 6 or 16 h. Cells were incubated with pan-caspase inhibitor ZVAD-fmk (20 μM) for 3 h prior to DZNep treatment. Protein transfer was confirmed by ponceau S staining, and equal loading was confirmed by GAPDH. (**B**) Apoptosis induction determined by subG1 analysis of cells pre-treated with DZNep (1 μM, 24 h) and treated with TRAIL (10 ng/ml, 16 h); ZVAD-fmk (20 μM) was given for 3 h prior to DZNep. (**C**) Activation of the mitochondrial apoptosis pathway was determined by TMRM staining for mitochondrial membrane potential (Δψ_m_) in Z-138 cells treated with DZNep (0.2, 1, 3, or 5 μM) alone for 40 h. (**D, E**) Analysis of Δψ_m_ of cells (Z-138, BJAB, and SU-DHL-9) treated with DZNep (1 μM, 24 h) followed by TRAIL (10 ng/ml for 1–5 h or 20 ng/ml for 16 h). Shown are percentages of cells with decreased Δψ_m_.

Following the observation of enhanced TRAIL-induced apoptosis in DZNep-pre-treated NHL-derived cell lines, we investigated the activation of caspases. Protein extracts of cells pre-treated with DZNep were incubated with TRAIL and analyzed for processing of caspases and PARP cleavage. In agreement with the increased DNA fragmentation (Fig. 1A), we detected enhanced cleavage/activation of caspases 8 and 3 in DZNep-pre-treated cells compared with cells treated with TRAIL alone (Fig. 3A). The JVM-2 cell line did not show any caspase activation coinciding with high cFLIP and low TRAIL receptor expression. Incubation with a pan-caspase inhibitor, ZVAD-fmk, prior to stimulation with DZNep and TRAIL stimulation, decreased cleavage of both caspase-8 and caspase-3, PARP a downstream target of caspases, as well as DNA fragmentation (Fig. 3A–B). These results indicate that DZNep-mediated enhancement of TRAIL-induced apoptosis depends on activation caspases. The enhanced cleavage of caspase-8 further suggests that the effect of DZNep in NHL occurs at or upstream of the caspase-8 activation step.

**Fig 4:**
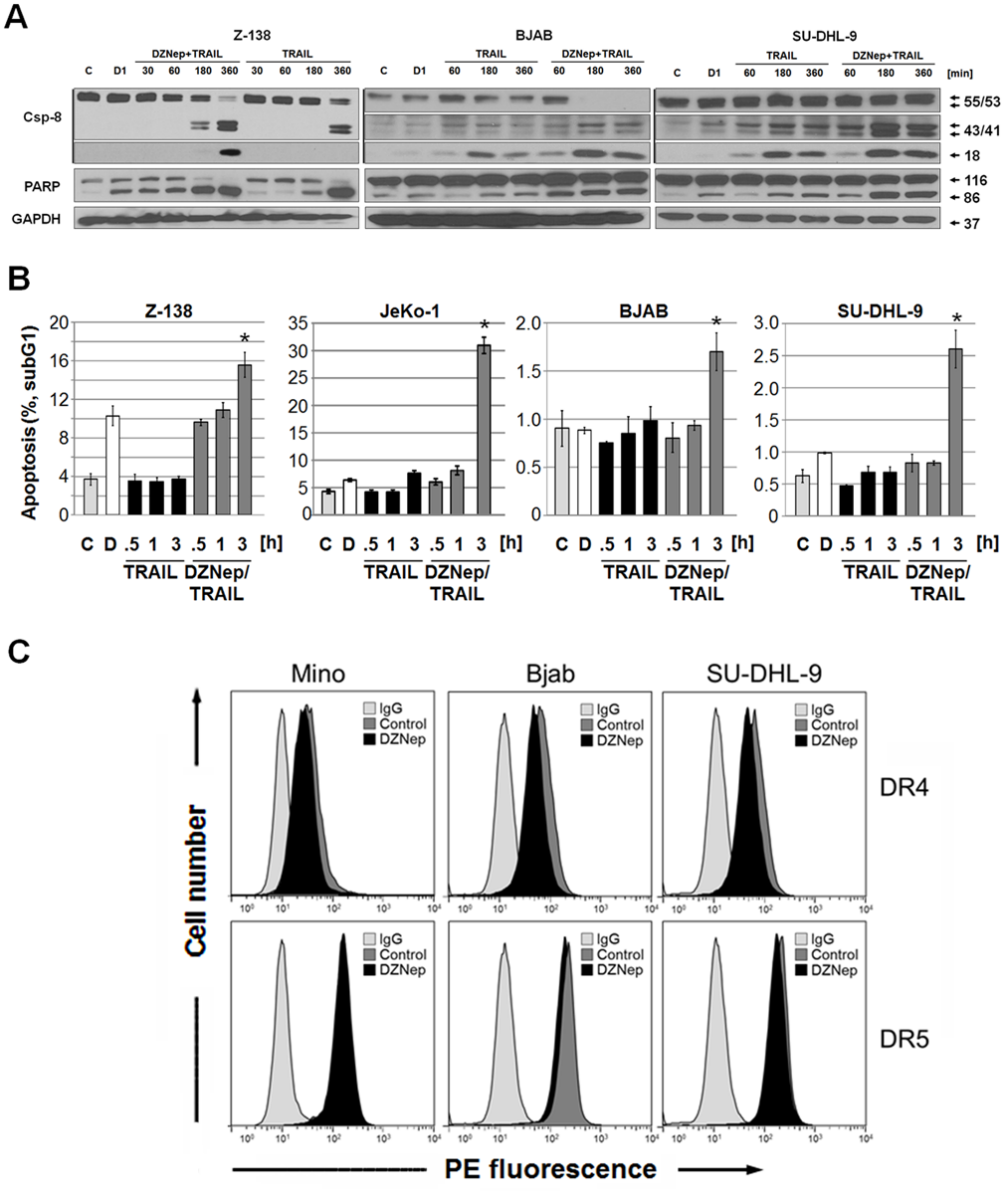
Early and accelerated processing of caspase-8 with DZNep treatment in lymphoma cell lines. (**A**) Cells pre-treated with DZNep (1 μM, 24 h) were stimulated with TRAIL (20 ng/ml, 0.5–6 h), and caspase-8 processing and PARP cleavage were analyzed by Western blotting. Protein transfer was confirmed by ponceau S staining, and equal loading was confirmed by GAPDH. (**B**) Apoptosis was determined by subG1 analysis for cells pre-treated with DZNep (1 μM, 24 h) followed by TRAIL (20 ng/ml, 0.5–3 h) stimulation. (**C**) TRAIL receptor (DR4 and DR5) surface expressions in DZNep-treated cells (1 μM, 24h) were analyzed by flow cytometry and compared with an IgG control. Shown are histograms of mean fluorescence (FL-3) of a representative experiment repeated twice.

The activation of the intrinsic mitochondrial apoptosis pathway was evaluated by changes in the mitochondrial membrane potential (Δψ_m_). Increasing doses of DZNep alone reduced the Δψ_m_ in Z-138 cells (Fig. 3C) whereas combined TRAIL and DZNep treatment led to greater Δψ_m_ impairment than did TRAIL alone, even with shorter TRAIL incubation times (Fig. 3E–D). To further delineate the impact of DZNep on TRAIL signaling, we analyzed apoptosis induction, caspase activation and cleavage of the caspase target PARP in TRAIL treated cells at shorter incubation intervals (0.5–6 h; Fig. 4A–B). Caspase-8 cleavage/activation was greater in DZNep-pre-treated cells than in cells treated with TRAIL alone, further confirming DZNep-induced removal of caspase-8 inhibition (Fig. 4A). In agreement with previous results, we detected substantially more DNA fragmentation but at lower magnitudes as expected with shorter incubation time in cells stimulated with both DZNep and TRAIL than in cells treated with TRAIL alone (Fig. 4B).

### DZNep-mediated reduction of cFLIP correlates with enhanced TRAIL-induced apoptosis

Surface expression of TRAIL receptors is a prerequisite for TRAIL signaling. We did not observe any significant changes in the surface expression of TRAIL death receptor 1 (DR4) or 2 (DR5) in cells incubated with 1 μM DZNep for 24 h compared with untreated controls (Fig. 4C).

**Fig 5:**
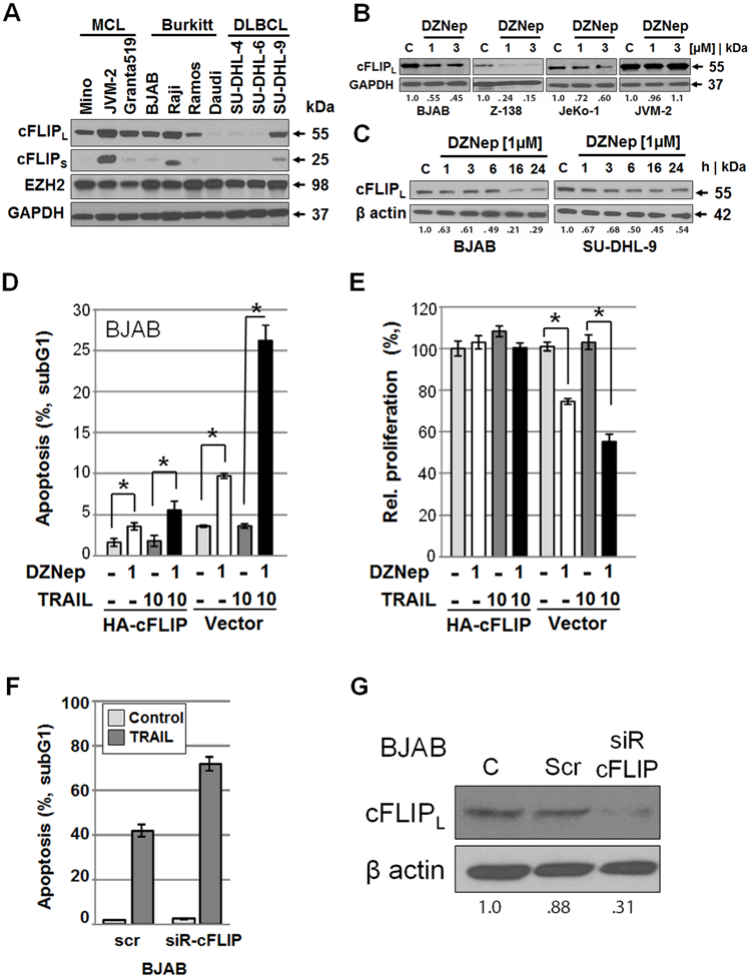
DZNep-mediated enhanced apoptosis correlates with reduced cFLIP expression levels. (**A**) Expression of cFLIP isoforms (long and short) and methyltransferase EZH2 in different non-treated cell lines by Western blotting. (**B, C**) cFLIP expression in cells treated with DZNep (1, 3 μM) for 24 h or with DZNep (1 μM) for 1–24 h. Protein transfer was confirmed by ponceau S staining, and equal loading was confirmed by GAPDH or β-actin. (**D, E**) Apoptosis induction and cell proliferation of BJAB cells transfected with an HA-cFLIP-expressing vector or a control vector followed by DZNep and TRAIL treatments as indicated are shown. (**F, G**) BJAB cells transfected with anti-cFLIP siRNA (siR) or scramble control siRNA (scr) followed by TRAIL. Reduced cFLIP level mediated by siRNA were analyzed by western blotting and equal loading was confirmed by β-actin.

These results combined with the accelerated cleavage of caspase-8 observed in DZNep- and TRAIL-stimulated cells pointed to DZNep impacting the death-inducing signaling complex. Thus, we investigated cFLIP expression levels in DZNep-treated cells (Fig. 5A). cFLIP levels were markedly decreased by DZNep treatment in a dose- and incubation time-dependent manner, particularly in Z-138 cells, whereas no changes were observed in JVM-2 cells that do not respond to TRAIL or the combination (Fig. 5B–C). As expected, DZNep pre-treatment of BJAB cells transfected with an HA-cFLIP-expressing vector substantially reduced TRAIL-induced apoptosis rates (Fig. 5D) as well as restored cell proliferation (Fig. 5E) compared to cells transfected with a control vector, indicating that cFLIP protected against DZNep-mediated enhancement of TRAIL-based killing. On the other hand suppression of cFLIP expression using cFLIP-targeting siRNAs enhanced sensitivity of cells to TRAIL-induced cell death (Fig. 4F–G). All these data point to the importance of cFLIP in inhibition of extrinsic apoptosis in NHL-derived cell lines.

### DZNep compromises cFLIP mRNA and cFLIP protein stability

**Fig 6:**
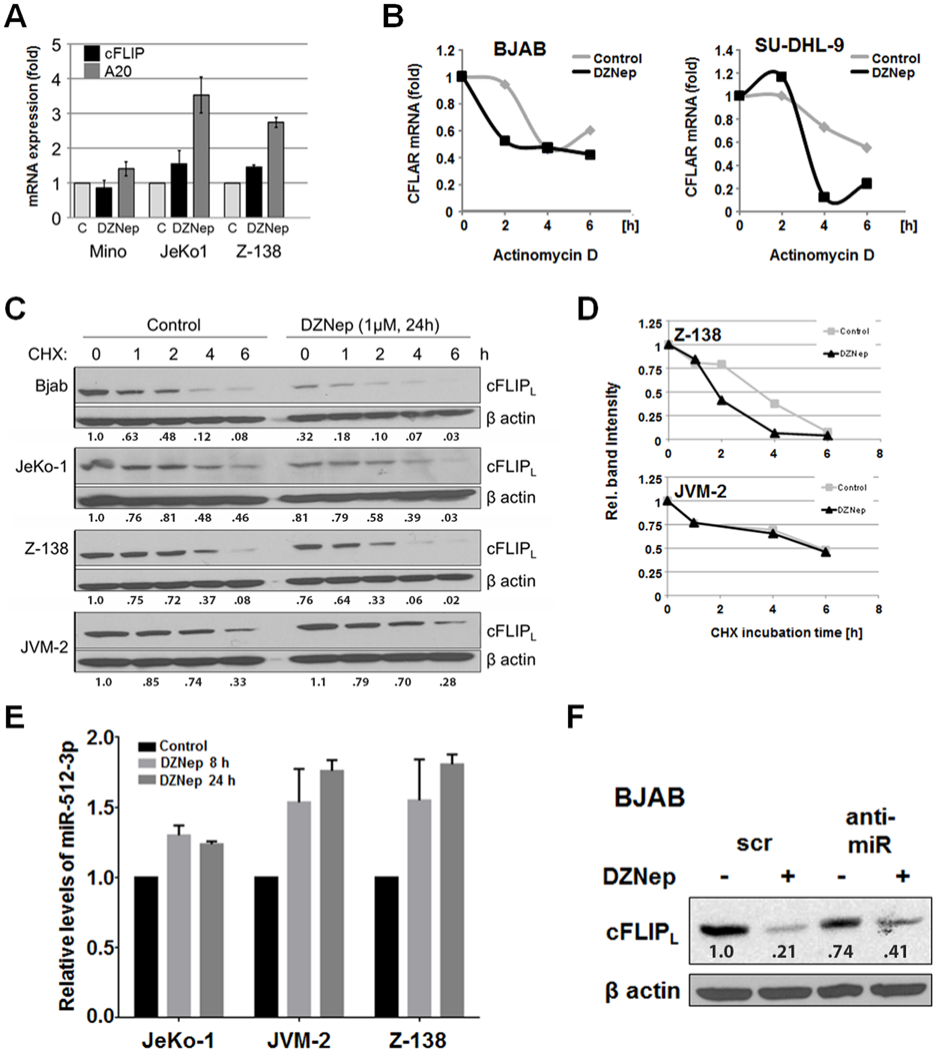
DZNep destabilizes cFLIP mRNA and accelerates cFLIP protein degradation. (**A**) cFLIP and A20 mRNA expression in DZNep-treated cells (1 μM, 24 h) analyzed by qRT-PCR. Data are shown as mRNA expression values relative to a control. (**B**) Analysis of cFLIP mRNA expression determined by qRT-PCR of cells treated with DZNep (1 μM) for 24 h followed by incubation with actinomycin D (2.5 μM, 2–6 h). (**C, D**) Expression level of cFLIP analyzed by Western blotting of cells treated with DZNep (1 μM) for 24 h followed by incubation with cycloheximide (50 μM, 0.5–6 h). Quantification of cFLIP expression by densitometry using ImageJ. (**E**) Relative expression levels of miRNA-512–3p in cells incubated with DZNep (1 μM) for 8 or 24 h analyzed by qRT-PCR. (**F**) cFLIP expression levels in DZNep-treated (1 μM, 24 h) BJAB cells after transfection with anti-miR-512–3p and anti-miR-346. Protein transfer was confirmed by ponceau S staining, and equal loading was confirmed by total β-actin.

Expression of cFLIP is regulated by NF-κB signaling, constitutive activation of which results in robust pro-proliferative and anti-apoptotic signaling in NHL cells [13]. We therefore investigated whether DZNep affects nuclear localization/activation of NF-κB subunits (p50 and p65) and thus reduces NF-κB-regulated gene expression. DZNep treatment did not significantly affect p50 or p65 translocation (data not shown) or the expression of cFLIP mRNA (Fig. 6A). Although cFLIP mRNA expression was not reduced by DZNep treatment, investigated cFLIP mRNA stability, using actinomycin D to block transcription, revealed impaired cFLIP mRNA stability in DZNep-treated cells compared to buffer treated controls (Fig. 6B). However DZNep treatment increased the transcription of another NF-kB target, A20, here used as a control. Incubation of cells with the translation inhibitor cycloheximide (CHX) revealed a lower cFLIP protein half-life in DZNep-treated cells than in controls (Fig. 6C–D). These results suggest that DZNep targets cFLIP expression through mRNA destabilization and enhances cFLIP protein degradation independently of NF-kB activation.

To elucidate the mechanism of DZNep-mediated effects, we focused on microRNAs known to be associated with regulation of cFLIP expression. DZNep-treated cells showed elevated levels of miR-512–3p (Fig. 6E), which has been reported to decrease cFLIP expression [10]. Transfection of DZNep-treated BJAB cells with miR-512–3p and miR-346 specific anti-microRNAs partially blocked DZNep-mediated cFLIP degradation (Fig. 6F). In addition, targeting of DICER expression increased cFLIP levels (data not shown) further supporting that microRNAs are involved in regulation of cFLIP expression. Thus, miR-512–3p, miR-346, and possibly additional microRNAs as well as additional mechanisms contribute to DZNep-mediated cFLIP mRNA degradation.

### Elevated levels of cFLIP mRNA in NHL patient samples

**Fig 7:**
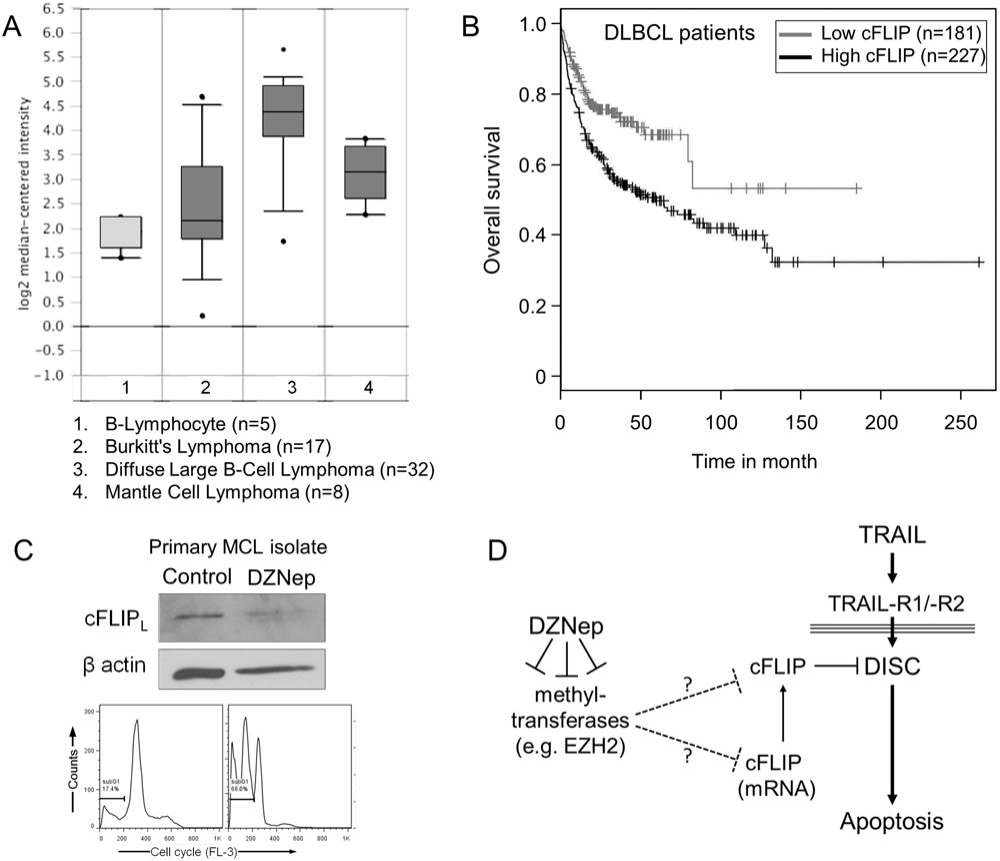
Expression of cFLIP mRNA in B lymphocytes and different NHL cells. (**A**) cFLIP mRNA expression in B lymphocytes, MCL, Burkitt and DLBCL cells (Oncomine database; www.oncomine.org, Reporter: 1867_at). (**B**) Kaplan-Meyer survival curve for DLBCL patients separated according to cFLIP expression (NCBI GEO ID: GSE10846). (**C**) cFLIP expression of MCL patient cells treated with DZNep (1 μM for 5 days) and apoptosis induction according to subG1 analysis. (**D**) Illustration of the presumed impact DZNep treatment has on TRAIL signaling by affecting cFLIP expression at different levels.

To further substantiate the importance of cFLIP in NHL cells, we analyzed gene expression data of NHL samples in the Oncomine database (Reporter: 1867_at) and found elevated levels of cFLIP mRNA in several NHLs compared to B-lymphocytes (Fig. 7A) [6]. Analysis of survival data from the NCBI Gene Expression Omnibus data set (GSE10846) of DLBCL [8, 25] patients revealed a worsening prognosis with elevated cFLIP mRNA levels (Fig. 7B) [2, 3]. In a primary MCL sample, treatment with DZNep alone significantly reduced cFLIP expression and induced apoptosis similar to results in NHL cell lines (Fig. 7C). These data show that NHL-derived cell lines as well as patient samples have increased levels of cFLIP and are potentially sensitized to apoptosis by DZNep-mediated cFLIP degradation.

## Discussion

Our results indicate that inhibition of methyltransferase activity leads to destabilization of cFLIP mRNA and enhanced degradation of cFLIP protein in NHL-derived cell lines. Reduced cFLIP protein levels sensitize cells to TRAIL-induced apoptosis through enhanced and accelerated TRAIL signaling.

Selective tumor cell toxicity makes TRAIL a promising cancer therapy target. Several clinical studies have shown that TRAIL is well tolerated but lacks desired efficacy as a single agent [4, 5, 18, 30], clearly underscoring the need for combination therapies that will enhance or enable TRAIL signaling in apparently resistant cells. Pan-histone deacetylase inhibitors, proteasome inhibitors and methyltransferase inhibitors, have been reported to enhance TRAIL-induced cell killing in different cancers [1, 12, 23]. However, the exact mechanism of sensitization to TRAIL remained elusive.

Resistance to death ligand (e.g., TRAIL and FasL)-mediated apoptosis and increased cFLIP expression are commonly encountered in NHL. In this study, we found that pre-treating cells with pan-methyltransferese inhibitor DZNep enhanced TRAIL-induced cell killing by reducing the levels of cFLIP. We investigated the potential mechanism in more detail. It is well established that cFLIP expression is regulated by NF-κB signaling, and inhibition of NF-κB is associated with enhanced TRAIL-mediated cell killing [37, 40]. DZNep did not interfere with the activation of NF-κB subunits (p50 or p65) in our study. Methylation marks on histones and non-histone proteins i.e. ERα, RB, STAT3, and p53 are important for transcription and for mRNA and protein stability. Although, DZNep could alter methylation of the cFLIP promoter and thus affect cFLIP transcription [33, 51], no significant reduction in cFLIP mRNA transcripts was observed in DZNep-treated cells. However, we detected a clearly shortened cFLIP mRNA half-life in DZNep-treated cells indicating a role for Alu elements present in cFLIP mRNA, which are known to form double-stranded RNA with a potential to decrease mRNA stability [49]. Binding and regulation of cFLIP mRNAs by RNA-binding proteins might be also influenced by methylation patterns [34, 44]. Altered methylation patterns presumably depend on the cell type and the respective cell context and may vary between different cells, but changes in methylation introduced by DZNep will likely affect multiple signaling processes [21, 56, 58, 60].

Both c-FLIP_L_ and c-FLIP_S_ isoforms are short-lived proteins that are degraded by the ubiquitin-proteasome degradation system initiated by poly-ubiquitination involving E3 ubiquitin ligases, some of which are shown to be repressed by methyltransferase activity [40, 59, 62]. Although we observed decreased half-life of cFLIP, we did not see a consistent increase in cFLIP ubiquitination in DZNep-treated NHL-derived cell lines. In addition, under oxidative stress, cFLIP has been shown to be the subject of phosphorylation leading to its subsequent degradation, adding to the complexity of cFLIP regulation [40, 52]. Although DZNep was shown to increase ROS levels in AML cells, we did not observe any significant ROS increase (data not shown) [61].

MicroRNAs were shown to target mRNAs of apoptosis-related proteins such as cFLIP [15, 16]. cFLIP-targeting microRNAs, miR-512–3p and miR-375, were recently reported to enhance paclitaxel-, and TNF-α–induced apoptosis, respectively [10, 48] In agreement with these previous findings, we observed increased levels of miR-512–3p in DZNep-treated cells. However, inhibition of miR-512–3p and miR-346 only partially blocked DZNep-mediated cFLIP degradation indicating that additional miRNAs and/or other mechanisms contribute to DZNep-mediated effects on cFLIP levels.

In summary, our results indicate that DZNep-mediated inhibition of methyltransferase activity destabilizes cFLIP mRNA and cFLIP protein in lymphoma cell lines. As a result of decreased cFLIP expression levels, cells displayed enhanced and accelerated TRAIL signaling, resulting in significantly increasing cell death. Although our data indicate that increased cFLIP ubiquitination and increased levels of microRNAs appear to be involved in regulation of cFLIP levels, the exact mechanisms underlying DZNep activity are yet to be determined (Fig. 7D). Given the effects of DZNep treatment on cFLIP levels in a broad range of NHL cells tested here, we anticipate that TRAIL-based therapies will benefit from combination with DZNep or other methyltransferease inhibitors. Taken together, our data provide a rationale for further investigation of the potential of methyltransferase inhibitors to improve TRAIL-based therapies in clinical models.

## Materials and Methods

### Cells and reagents

Cells from mantle cell lymphoma (MCL)-patients were collected under The University of Texas MD Anderson Cancer Center IRB-approved protocol Lab08–0190 with the written consent of patients. Primary MCL cells were propagated on a pre-established layer of human bone marrow mesenchymal stromal cells (hMSC; Lonza, Allendale, NJ,USA) (10:1 MCL to stromal cell ratio) using hMSC media supplemented with mesenchymal cell growth factors and glutamine (Lonza) at 37°C in 5% CO_2_. For treatment, MCL cells were harvested and resuspended in 50% fresh and 50% conditioned media from hMSC cultures.

Cell lines derived from mantle cell lymphoma (Mino, JeKo-1, Z-138, and JVM-2), Burkitt’s lymphoma (BJAB, Raji, Ramos, and Daudi), and diffuse large B-cell lymphoma (SU-DHL-4, SU-DHL-6, and SU-DHL-9) were obtained from ATCC (Manassas, VA, USA). Cell lines were authenticated by short tandem repeat analysis at the Characterized Cell Line Core Facility at The University of Texas MD Anderson Cancer Center, Houston, TX, USA and regularly tested for mycoplasma (Lonza). All cell lines were grown in Roswell Park Memorial Institute 1640 (RPMI 1640) medium (Thermo Scientific HyClone, Logan, UT, USA) supplemented with 10% heat-inactivated fetal bovine serum (Atlanta Biologicals, Atlanta, GA, USA) and 2 mmol/l glutamine (Life Technologies-Invitrogen, Carlsbad, CA, USA) at 37°C in a humidified atmosphere with 5% CO_2_. The medium was changed twice weekly. Treatments with the indicated agents were initiated 24 h after seeding cells. Cells were treated with 0.2–5 μM DZNep (Cayman Chemicals, Ann Arbor, Michigan) 24 h-5 days; 10–20 ng/ml of *Killer*TRAIL (Alexis Biochemicals, Gruenberg, Germany) for 0.5–16 h; 20 μM pan-caspase inhibitor ZVAD-fmk (R&D Systems, Minneapolis, MN, USA) applied 3 h before DZNep treatment; 50 μg/ml of cycloheximide (Sigma-Aldrich, St. Louis, MO, USA) for 2–6 h; and 2.5 μM actinomycin D (Sigma-Aldrich) for 2–6 h.

### Evaluation of apoptosis, necrosis, and proliferation

Apoptosis was determined by the size of the subG1 cell population [36]. Briefly, cell pellets were stained with a hypotonic solution containing 40 μg/ml of propidium iodide, 0.1% sodium citrate, and 0.1% Triton X-100 for 2 h. The DNA content in cell nuclei was subsequently analyzed with a flow cytometer (FACSCalibur; BD Biosciences, San Diego, CA, USA) and FlowJo software (Tree Star, Ashland, OR, USA). Necrosis was measured by monitoring lactate dehydrogenase levels in cell supernatants (Cytotoxicity Detection Kit (LDH); Roche Diagnostics, Madison, WI). Cell viability was determined by the esterase activity evaluated by staining with 1 μM calcein AM (Invitrogen) for 30 min at 37°C. To assess cell proliferation, cells were seeded in 96-well plates (10,000 cells per 100 μl),mitochondrial enzyme activity was evaluated using CellTiter 96 AQueous Non-Radioactive Cell Proliferation Assay (Promega, Madison, WI, USA), and absorbance at 450 nm was measured by a VICTOR 3V plate reader (PerkinElmer, Waltham, MA, USA). Data were analyzed and reported as percentages of untreated controls.

### Western blot analysis

Cells were harvested by centrifugation, washed with ice-cold phosphate-buffered saline (PBS) and lysed with RIPA cell lysis buffer (Cell signaling, Danvers, MA, USA). Protein concentration was determined by bicinchoninic acid assay (BioRad, Hercules, CA, USA) according to the manufacturer’s protocol. Primary antibodies purchased from Cell Signaling Technology were caspase-8 (#9746; 1:1,000), cleaved caspase-3 (#9661; 1:1,000), and poly (ADP-ribose) polymerase (PARP; #9542; 1:1,000). cFLIP antibodies (sc-5276, 1:200) were obtained from Santa Cruz Biotechnology (Santa Cruz, CA, USA). β-actin—horseradish peroxidase (Sigma-Aldrich, 1:40,000) and glyceraldehyde 3-phosphate dehydrogenase (GAPDH) (GeneTex, Irvine, CA, USA; GTX100118, 1:20,000) antibodies were used to compare loading of individual lanes. Horseradish peroxidase—labeled goat anti-rabbit and goat anti-mouse secondary antibodies (1:5,000) were obtained from Jackson Laboratory (Bar Harbor, ME, USA). Intensity of protein bands were quantified through densitometry using Image J software (National Institutes of Health, Bethesda, MD, USA).

### Mitochondrial membrane potential (Δψ_m_)

To determine Δψ_m_ by flow cytometry, cells were harvested, washed with PBS, and stained with 1 μM fluorescent tetramethylrhodamine methyl ester perchlorate (TMRM+; Sigma-Aldrich) for 15 min at 37°C. The amount of dye in the mitochondria was evaluated by flow cytometry (FACSCalibur; BD Biosciences) and analyzed by FlowJo software (Tree Star).

### TRAIL receptor (DR4 and DR5) surface expression

Surface expression levels of TRAIL receptors were analyzed by flow cytometry. Cells were harvested by centrifugation and washed with PBS. Aliquots of 1x10^6^ cells in 100 μl of PBS with 1% bovine serum albumin were incubated for 30 min in the dark with phycoerythrin-conjugated antibodies to TRAIL receptors DR4/TRAIL-R1 or DR5/TRAIL-R2 (#307205 or #307405, BioLegend, San Diego, CA, USA). Phycoerythrin-labeled isotype IgG1 antibody (clone MOPC 31C, BioLegend) was used as a negative control.

### RNA isolation and quantitative real-time polymerase chain reaction (qRT-PCR)

Total RNA was extracted by column centrifugation using RNeasy kit (QIAGEN Sciences, Rockville, MD) according to the manufacturer’s instructions. First-strand complementary DNA was synthesized using a SuperScript II reverse transcriptase kit (Invitrogen-Life Technologies) according to the manufacturer’s protocol. Samples were analyzed on 96-well microtiter plates using the StepOnePlus Real-Time PCR System (Applied Biosystems-Life Technologies) with cFLIP, A20, and GAPDH TaqMan probes (Applied Biosystems) after 40 cycles at 95°C for 15 sec followed by 60°C for 1 min. StepOne software version 2.1 (Applied Biosystems) was used to analyze the qRT-PCR data as described previously [41]. MicroRNA isolation from the samples was performed using mirVana isolation kit (Ambion-Life Technologies) per the manufacturer’s protocol. The TaqMan MicroRNA Assays target-specific stem-loop reverse transcription primers were used for amplification of mature microRNAs followed by standard TaqMan Assay-based real-time PCR.

### Transfection

Cells were transfected using the Neon Transfection System (Life Technologies) according to the manufacturer’s protocol. BJAB cells were transfected with an empty pcDNA3-vector or with an HA-tagged cFLIP—expressing vector (provided by S. Matsuzawa and J.C. Reed) and assayed 24 h later. BJAB cells were transfected with scrambled or with cFLIP-targeting siRNA (ON-TARGETplus, Dharmacon, Pittsburgh PA, USA) prior to incubation with TRAIL or non-targeting microRNA or with anti-miR-512–3p and anti-miR-346 (Invitrogen) prior to incubation with DZNep. Expression of cFLIP in transfected cells was determined by Western blotting.

### Statistical analysis

Experimental data are reported as means ± standard deviations (SDs) from a representative experiment performed in triplicate. Differences between groups were calculated using a two-tailed Student *t*-test. *P* < 0.05 was considered statistically significant and indicated by an asterisk (*).
